# An Attenuated Targeted-TNF Localizes to Tumors In Vivo and Regains Activity at the Site of Disease

**DOI:** 10.3390/ijms221810020

**Published:** 2021-09-16

**Authors:** Sheila Dakhel, Christian Lizak, Mattia Matasci, Jacqueline Mock, Alessandra Villa, Dario Neri, Samuele Cazzamalli

**Affiliations:** 1 Philochem AG, CH-8112 Otelfingen, Switzerland; Sheila.Dakhel@philochem.ch (S.D.); Christian.Lizak@philogen.com (C.L.); Mattia.Matasci@philogen.com (M.M.); Jacqueline.Mock@philochem.ch (J.M.); Ale.mvilla@gmail.com (A.V.); dario.neri@pharma.ethz.ch (D.N.); 2 Philogen S.p.A., Piazza La Lizza, 7, 53100 Siena, Italy

**Keywords:** tumor targeting, immunocytokines, activity-on-demand, targeted-tumor necrosis factor, EDB-fibronectin

## Abstract

Antibody-cytokine fusion proteins (immunocytokines) are gaining importance for cancer therapy, but those products are often limited by systemic toxicity related to the activity of the cytokine payload in circulation and in secondary lymphoid organs. Tumor necrosis factor (TNF) is used as a pro-inflammatory payload to trigger haemorrhagic necrosis and boost anti-cancer immunity at the tumor site. Here we describe a depotentiated version of TNF (carrying the single point mutation I97A), which displayed reduced binding affinity to its cognate receptor tumor necrosis factor receptor 1 (TNFR-1) and lower biocidal activity. The fusion of the TNF(I97A) mutant to the L19 antibody promoted restoration of anti-tumor activity upon accumulation on the cognate antigen, the alternatively spliced EDB domain of fibronectin. In vivo administration of high doses (375 μg/Kg) of the fusion protein showed a potent anti-tumor effect without apparent toxicity compared with the wild type protein. L19-TNF^I97A^ holds promise for the targeted delivery of TNF activity to neoplastic lesions, helping spare normal tissues.

## 1. Introduction

Cytokines are small proteins which modulate the activity of the immune system in health and in disease. Certain proinflammatory cytokines are used for cancer immunotherapy [1,2], while other cytokines with anti-inflammatory properties may be considered as tools for the treatment of chronic inflammatory and autoimmune conditions [3]. Recombinant cytokine products on the market include interleukin-2 (IL-2) (Proleukin^®^), IL-11 (Neumega^®^), tumor necrosis factor (TNF; Beromun^®^), interferon (IFN)-α (Roferon A^®^, Intron A^®^), IFN-β (Avonex^®^, Rebif^®^, Betaseron^®^), IFN-γ (Actimmune^®^), granulocyte colony-stimulating factor (Neupogen^®^), and granulocyte macrophage colony-stimulating factor (Leukine^®^). While the use of cytokines can be quite effective in fit patients that tolerate systemic side effects, which often manifest even at sub-milligram doses [4,5,6], the escalation to therapeutically active regimens is usually hindered by the onset of limiting toxicity. Common side effects associated with proinflammatory cytokines include fever and chills, hypotension, fatigue and vascular leak syndrome [7,8,9,10].

Targeting an active cytokine to a tumor-associated antigen by means of a fusion with a suitable monoclonal antibody may represent a strategy for the enhancement of the cytokines’ therapeutic index [11,12]. The strategy relies on the observation that tumor resident T cells may be enriched for cancer specificity when compared to secondary lymphoid organs [13]. Various immunocytokine products have been tested in preclinical models and in cancer patients, both as monotherapy and in combination with other treatments such as conventional chemotherapy [14], naked antibodies [15], radiation [16], immune check-point inhibitors [17] and other immunocytokines [18,19].

Tumor necrosis factor (TNF) is a homotrimeric cytokine which shows direct cytotoxic activity on certain types of cancer cells [20], stimulates anti-tumor immunity [21,22], and may also be active on the tumor endothelium, leading to intravascular blood coagulation [23] or to haemorrhagic necrosis [24]. Recombinant human TNF (Beromun^®^) is an approved drug in Europe for the treatment of metastatic melanoma and soft tissue sarcoma in the setting of isolated limb perfusion. The systemic use of non-targeted TNF is limited by its narrow therapeutic index and high toxicity at very low doses [25]. Therefore, TNF has been used as an active payload for the generation of tumor-specific immunocytokines [26].

L19-TNF (Fibromun, Philogen S.p.A., Siena, Italy) is a fully-human immunomodulatory product consisting of a non-covalent homotrimeric immunocytokine featuring human TNF fused to the L19 antibody (specific to EDB of fibronectin, a marker of tumor neovasculature) [12]. L19-TNF has been systemically administered to patients with solid tumors [27], as well as in isolated limb perfusion procedures to patients with in-transit melanoma metastases [28]. Currently, Fibromun is being investigated in combination with doxorubicin or dacarbazine for the treatment of soft tissue sarcoma (EudraCT number: 2016-003239-38, NCT03420014 and EudraCT:2018-004104-19). Moreover, phase I/II studies have recently being started in newly diagnosed and in recurrent glioblastoma patients (NCT03779230, NCT04443010 and NCT04573192).

A promising strategy to further enhance the therapeutic index of cytokines consists in the design of products which regain full activity upon accumulation at the tumor site [29,30]. The use of cytokine payloads with decreased affinity towards the cognate receptor lowers the biological activity of the molecule in systemic circulation, thus allowing the administration of higher systemic doses. Promising preclinical results have been obtained with certain antibody-cytokine fusions based on a depotentiated version of IFNα, IL12 or 4-1BB [31,32,33,34]. Moreover, the group of Tavernier have developed Activity-on-Target cytokines (so called Actakines) by fusing inactivated TNF or IFN-γ mutants to a CD13 antibody, leading to selective targeting to the tumor endothelium and complete tumor destruction without side-effects [35].

In this article, we present the generation of a depotentiated version of Fibromun product, capable of inducing potent in vivo anti-tumor activity with reduced systemic toxicity. The new molecule, named L19-TNF^I97A^, was selected among a series of candidates displaying single-point mutations of amino acid residues involved in surface interactions between TNF monomers. The substitution of isoleucine for alanine in position 95 of the human TNF, hence named L19-TNF^I97A^, showed decreased activity (~100-fold) when compared to wild type L19-TNF (L19-TNF^WT^), while its biological activity was restored upon binding to the target antigen EDB both in vitro and in vivo. Moreover, the binding affinity of L19-TNF^I97A^ towards its cognate receptor, TNFR1, was decreased compared to the wild type molecule. Preclinical characterization of L19-TNF^I97A^ in tumor-bearing mice revealed a potent anti-tumor effect with negligible systemic toxicity. The use of engineered mutant versions of immunocytokines, like L19-TNF^I97A^, could represent a new avenue to develop products with ‘activity-on-demand’ properties.

## 2. Results

### 2.1. 3D-Structure Analysis of the Trimeric TNF Reveals Potential Mutations for Depotentiated Versions of the Molecule

**Figure 1:**
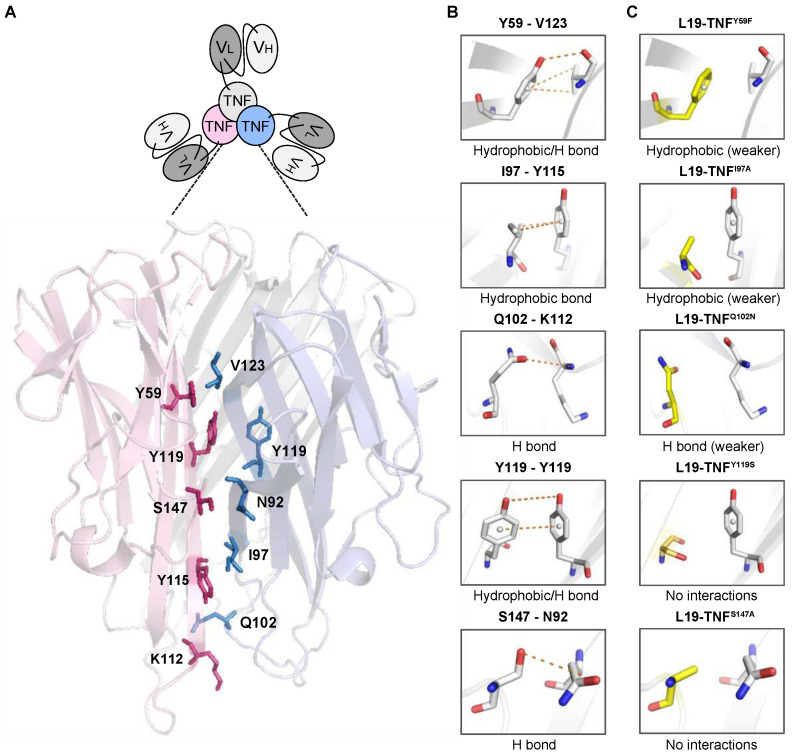
Graphical representation of the most relevant TNF monomer-monomer surface interactions in L19-TNF. (**A**) Scheme of the Fibromun product and 3D structure of the interaction surface between TNF monomers. (**B**) Side chains of amino acids involved in non-covalent interactions between TNF subunits. (**C**) List of mutations considered in this article and schematic 3D illustration of the single side chain interactions after mutation by design.

Soluble monomeric human TNF naturally forms a homotrimer which represents the active molecular entity and binds to TNF receptors, thus inducing a number of pro-inflammatory responses including cell cytotoxicity [36]. Our study was initially based on the identification of potential residues for the generation of TNF mutants which exhibit a decreased trimerization potential and, therefore, a reduced potency in solution. We reasoned that, upon antigen-dependent clustering, the biological activity of an immunocytokine featuring a TNF mutant as payload would be restored selectively at the site of disease. After performing a structural analysis of TNF (PDB ID:1TNF) we identified amino acid residues at the monomer-monomer interface that are involved in trimerization [37]. Figure 1A represents the trimeric form of wild type TNF (TNF^WT^) in which selected amino acids for mutagenesis are shown in stick representation and labelled. We then predicted individual amino acid substitutions to abrogate or to weaken the formation of non-covalent interactions involved in trimerization (Figure 1B,C). Moreover, some of our selected residues for the generation of TNF mutants matched the work of Tavernier’s group, in which they described targeted single chain TNF variants with reduced activity to its receptor (US20160159874A1).

### 2.2. Production and Characterization of L19-TNF Mutant Fusion Proteins

**Figure 2:**
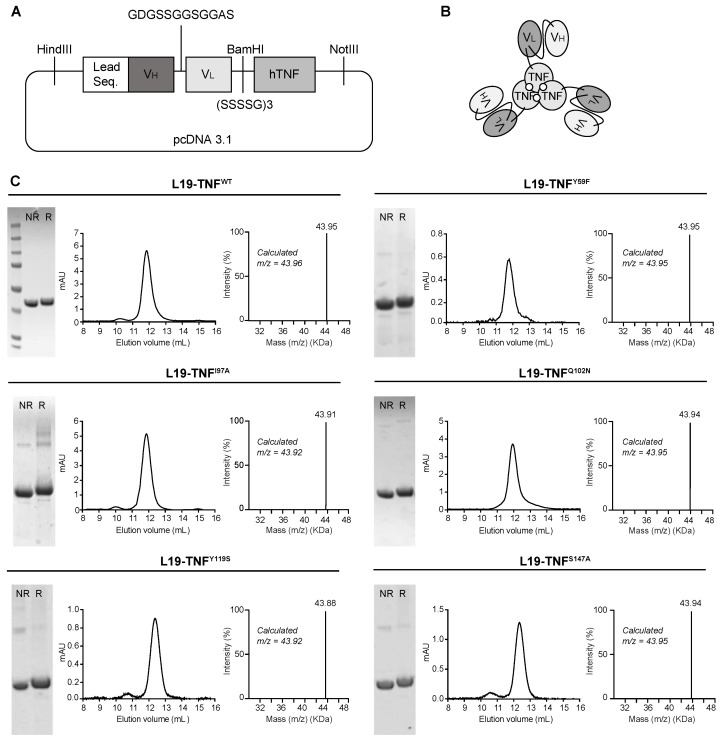
Cloning, expression and quality control analyses of L19-TNF mutants. (**A**) Schematic representation of L19-TNF^WT^ vector used as a template for the generation of mutants. Leader sequence: MGWSLILLFLVAVATGVHS (**B**) General scheme of L19-TNF mutants. (**C**) Quality control of L19-TNF^WT^ and of L19-TNF^Y59F^, L19-TNF^I97A^, L19-TNF^Q102N^, L19-TNF^Y119S^, L19-TNF^S147A^ mutants by SDS-PAGE, Size Exclusion Chromatography (pic eluting at 12 mL corresponding to the homotrimer form) and LC/MS analyses. NR = non reducing conditions, R = reducing conditions.

L19-TNF is a recombinant fusion protein composed by the fully human L19 antibody in scFv format conjugate to the human TNF by a (S_4_G)_3_ linker (Figure 2A) forming a natural homotrimer (Figure 2B). To identify products with promising features for subsequent in vivo investigations, 11 different mutant versions of L19-TNF were expressed in mammalian cells and purified by Protein A chromatography, as the VH domain of the L19 antibody is compatible with affinity capture modality [38]. Six of the eleven products exhibited favorable size exclusion and sodium dodecyl-sulfate polyacrylamide gel electrophoresis (SDS-PAGE) profiles (Figure 2C and Supplementary Information Figure S1). Deconvolution of the liquid chromatography-mass spectrometry (LC-MS) profile allowed identification of the expected experimental masses for all the targeted TNF-constructs which were compatible with the predicted values in the absence of any protein glycosylation. Sequences of the fusion proteins are listed in the Supplementary Information.

### 2.3. Conditional In Vitro Cytotoxicity Effect of L19-TNF Mutants upon EDB Concentration

**Figure 3:**
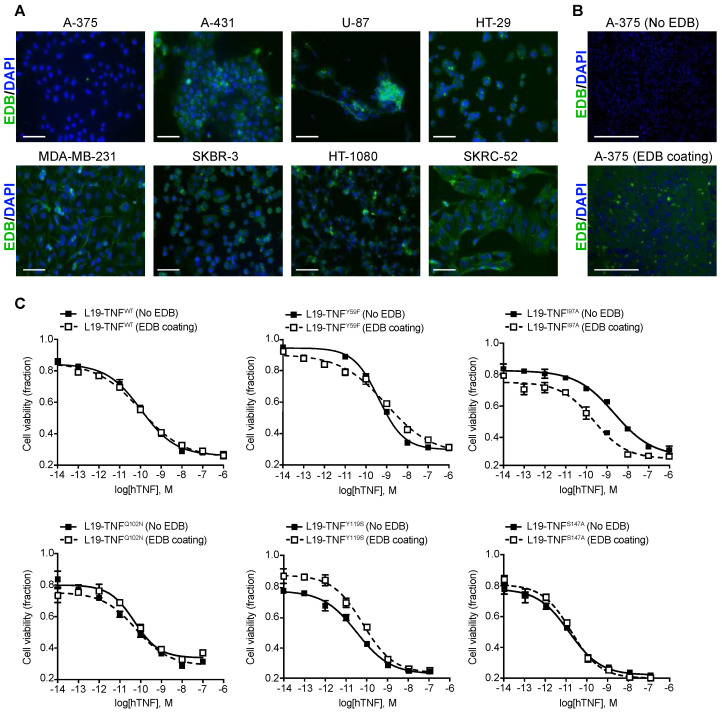
Assessment of EDB expression by different human tumor cell lines and in vitro biocidal activity of L19-TNF mutants. (**A**) Immunofluorescence analysis of EDB expression on different human tumor cells. All tumor cells showed high EDB expression, with the exception of the A-375 cell line. Green = EDB, Blue = Nucleus. Scale bar = 100 µm. (**B**) Immunofluorescence EDB staining results on A-375 cultured in absence or presence of recombinant EDB coating. (**C**) In vitro cytotoxicity of L19-TNF wild type and mutants assayed on A-375 cells in the presence (dashed line) or absence (continuous line) of EDB coating. L19-TNF^I97A^ showed conditional cytotoxicity in the presence of EDB. Data points represent cell viability (mean ± SD, *n* = 3).

TNF is known to induce direct killing on several tumor cell lines [24]. To evaluate the biocidal activity of L19-TNF muteins upon antigen concentration, a panel of human tumor cell lines were analyzed for the EDB expression by immunofluorescence (Figure 3A). The antigen was not detectable in the human melanoma cell line A-375 and, therefore, the L19-TNF mutants were tested for their cytotoxic activity on these cells in the presence or absence of EDB coating (Figure 3B). Cell killing of L19-TNF^WT^ and the majority of the mutants tested was not affected by the presence of EDB. However, in the absence of EDB, the mutein carrying the single substitution of the isoleucine at position 97 by alanine (hence named L19-TNF^I97A^) showed 10 times less potency compared to the wild type version. This effect was re-established upon EDB presence (Figure 3C). EC_50_ values for all the tested proteins are reported in Table 1 and Supplementary Information Table S4.

### 2.4. The Mutant I97A Has Reduced Binding Affinity towards TNFR1

**Figure 4:**
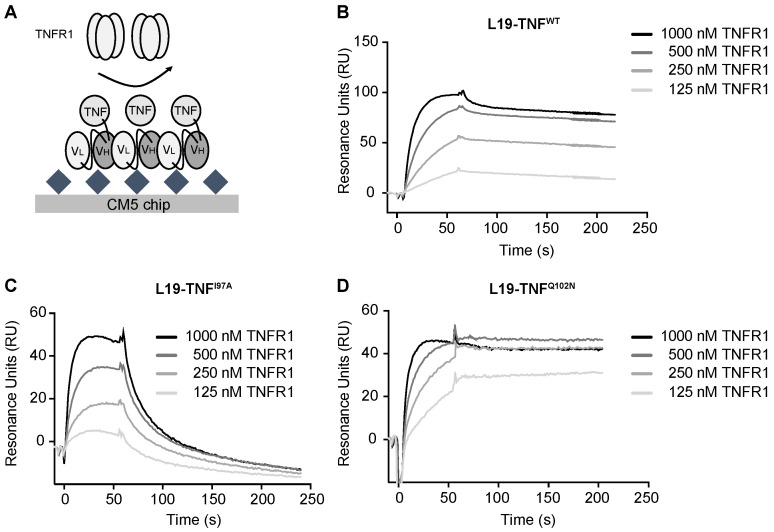
Real time binding analysis of TNFR1 on immobilized L19-TNF muteins. (**A**) Schematic representation of affinity measurement methodology. EDB was covalently immobilized on the chip, while different L19-TNF muteins were coated on the solid surface thanks to non-covalent L19-EDB interaction. TNFR1 was sequentially injected at different concentrations (starting concentration of 1000 nM, followed by 1:2 dilution series). SPR sensorgrams of (**B**) L19-TNF^WT^, (**C**) L19-TNF^Q102N^ and (**D**) L19-TNF^I97A^ are presented.

Affinity of L19-TNF^WT^ and of L19-TNF^I97A^ was analyzed by Surface Plasmon Resonance. The proteins were reversibly coated on independent EDB-precoated chips. TNFR1 was injected at different concentrations and SPR response was measured (Figure 4A). L19-TNF^WT^ showed a fast k_on_ and very slow k_off_ towards TNFR1 (experimental calculated KD = 10 nM) and the binding kinetics of the mutant L19-TNF^Q102N^ (selected as a mutant with similar in vitro activity to the wild type protein) were comparable (Figure 4B,D). By contrast, the L19-TNF^I97A^ mutant was still a stable homotrimer in gel filtration analysis but exhibited a faster dissociation from the receptor, indicative of a depotentiation of TNFR1 binding (Figure 4C). Fitting and calculated binding constants are depicted in the Supplementary Information Tables S5 and S6.

### 2.5. The I97A Mutant Accumulates at the Tumor Site and Shows High Levels in Blood

**Figure 5:**
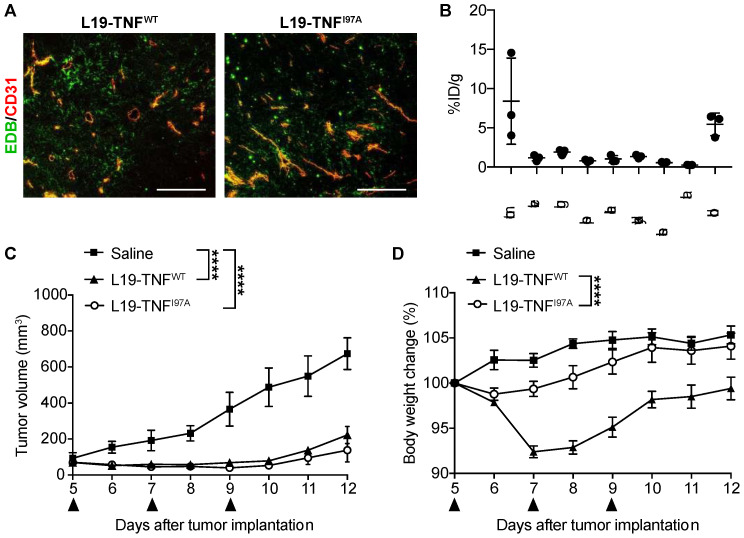
Tumor-targeting performance of L19-TNF^I97A^ and in vivo therapy study in tumor-bearing mice. (**A**) Immunofluorescence staining of WEHI-164 tumors with L19-TNF^I97A^ (L19-TNF^WT^ was used as the positive control of the experiment). Green = L19-TNF^WT^ or L19-TNF^I97A^, Red = CD31. Scale bar = 100 µm. (**B**) In vivo biodistribution with radioiodinated L19-TNF^I97A^ in BALB/c mice bearing WEHI-164 subcutaneous tumors, 24 h after intravenous administration. The product shows selective accumulation in tumor lesions (*n* = 3). Assessment of therapeutic efficacy and toxicity in BALB/c mice bearing WEHI-164 subcutaneous tumors. Mice were injected with a high dose of L19-TNF products (375 µg/kg, black arrows indicate IV administration). Tumor volume (**C**) and changes in animal body weight (**D**) induced by the administered therapeutic proteins were monitored throughout the study (mean ± SEM; *n* = 3). The statistical analysis for the tumor volume was performed using a two-way ANOVA analyses with Bonferroni post-test **** *p* < 0.0001).

In order to test immunoreactivity of the L19 antibody function and EDB in L19-TNF^I97A^, immunofluorescence staining was performed on non-treated WEHI-164 tumor cryosections. L19-TNF^WT^ and L19-TNF^I97A^ were both able to bind to EDB present in tumor specimens (Figure 5A). The tumor-targeting performance of L19-TNF^I97A^ was evaluated in immunocompetent BALB/c mice bearing subcutaneously-grafted WEHI-164 tumors. The protein was radiolabeled with ^125^I and injected intravenously at the recommended dose of 250 µg/Kg. The mutein preferentially localized at the site of the disease, with a high tumor uptake value and excellent tumor-to-normal organs ratio (average tumor-to-organs ratio 5.5:1). Surprisingly, high levels of the mutant were found in blood 24 h post-administration (average tumor-to-blood ratio of 2-to-1) (Figure 5B).

### 2.6. I97A Mutein Retains Anti-Tumor Efficacy While Decreasing Toxicity in Mice

Our group has previously shown that therapeutic index and anti-cancer activity of potent cytokines such as IL-2 [11,15], IL-12 [39] or TNF [12,40], can be improved by fusing them to the L19 antibody in various formats. In this study, the therapeutic activity and tolerability of the parental L19-TNF^WT^ was directly compared to the L19-TNF^I97A^ mutant in a syngeneic mice model study. WEHI-164 murine fibrosarcoma represents an established model in which anti-tumor activity and toxicity of TNF-based pharmaceuticals are commonly tested [26,41]. The maximal tolerated dose (MTD) of the fully human L19-TNF^WT^ product was found to be 375 μg/kg in mice (Supplementary Information Figure S2). Immunocompetent mice bearing subcutaneously-grafted WEHI-164 tumors were intravenously administered with L19-TNF^WT^, L19-TNF^I97A^ or saline (control of the experiment) and toxicity was evaluated by measuring daily changes in the body weight. While administration of L19-TNF^WT^ caused a reversible and significant body weight loss of 10%, treatment with L19-TNF^I97A^ led to potent anti-tumor efficacy (Figure 5C) with negligible systemic toxicity over the entire treatment (Figure 5D).

## 3. Discussion

In this article, we have described a strategy to minimize off-target toxicity related to the systemic administration of TNF-based therapeutics, which regain activity once having localized at the site of disease. We generated and tested a series of novel antibody-TNF fusion proteins using engineered TNF mutants with strongly reduced affinity to its receptor, with the aim of further enhancing the therapeutic index of the Phase III clinical-stage L19-TNF product.

TNF (Beromun^®^) is administered locally for the treatment of sarcoma patients by means of isolated limb perfusion procedures at 2–2.5 mg/m^2^ [41,42]. This dose range is far above the maximum tolerated dose of 150–400 μg/m^2^ which has been determined for systemic administration of the same product [41,42]. Antibody-TNF fusions are being developed clinically with the aim to increase efficacy and reduce off-target toxicity of the cytokine payload after systemic administration. L19-TNF is an homotrimeric fusion protein composed by the human TNF fused to the L19 antibody (specific for the alternatively spliced extra domain B of Fibronectin). The immunocytokine has shown to be safe up to 1 milli-gram per patient when administered by intravenous infusion [39]. While L19-TNF preferentially accumulates at the tumor site and exhibits an improved therapeutic window compared to recombinant TNF [12], the biological activity and tolerability of the two products are comparable [43]. L19-TNF has shown encouraging activity in patients suffering from metastatic soft tissue sarcoma or high-grade glioma [26,41]. It is reasonable to postulate that L19-TNF variants with an enhanced therapeutic index may further improve the anti-cancer activity of this class of drugs.

There is a growing interest in cytokine-based pharmaceuticals with “activity-on-demand” properties and a number of approaches have been considered to this end. For instance, the conditional reassembly of heterodimeric cytokines (i.e., IL-12 consisting of p40 and p35 subunits) at the tumor site has been reported after subsequent administration of targeted cytokine subunits [32]. Some receptors of the TNF superfamily rely on the formation of higher-order clusters for effective signaling. This feature was demonstrated in a recent study, where antibody fusions exploited the ability of 4-1BB ligand to oligomerize upon antigen binding and regain activity at the site of disease [31]. Multiple research groups have focused their efforts on the development of attenuated versions of cytokines to reduce toxicity. The generation of mutant versions of TNF and IFN-γ, with strongly reduced affinity to its receptor, while being fused to a CD13 single domain antibody has been recently reported [30,44]. The combination treatment with both immunocytokines led to selective apoptosis of the tumor endothelium and complete tumor eradication without side effects [32]. We expanded upon this strategy by specifically targeting attenuated human TNF to the tumor microenvironment via a clinical stage anti-EDB antibody named L19.

For preclinical in vivo evaluation of TNF-based products, although murine TNF is usually used as a surrogate molecule, the human payload can also be employed as it cross-reacts with the murine TNFR1 and triggers killing of mouse tumor cells [45,46]. In our study, the single point mutation I97A (located at the interface between TNF subunits) reduced the binding affinity of TNF to its cognate receptor TNFR1, resulting in an approximately 50-fold faster dissociation rate when compared to the wild type protein (Figure 4). The I97A mutation enabled dose escalation of the immunocytokine beyond the previously established MTD (i.e., 375 μg/Kg), with non-detectable toxicity in a preclinical model of cancer [40]. In contrast, the respective wild type TNF fusion protein showed lower tolerability when administered at an equimolar dose in the same tumor model (Figure 5). Despite strong attenuation of payload activity, L19-TNF^I97A^ retained full capacity to selectively accumulate at the tumor site, induce rapid necrosis, and have a potent anti-tumor effect in fibrosarcoma-bearing mice.

In summary, we described a novel, targeted TNF fusion protein that retains the potent in vivo anti-tumor activity of wild type TNF while reducing its systemic side effects. As the EDB domain of fibronectin is expressed in the majority of human malignancies and the L19 antibody has been shown to localize to tumors in human patients, the new product has the potential to provide a therapeutic benefit to a wide variety of cancers. The potent anti-tumor activity with reduced toxicity may provide L19-TNF^I97A^ a broader therapeutic index when compared to conventional immunocytokines based on wild type TNF. We anticipate that, in order to understand differences of TNFR1 activation in normal and malignant cells by the mutant L19-TNF^I97A^, additional mechanistic studies will be needed. Moreover, safety pharmacology studies in non-human primates will help to accurately assess the tolerability of L19-TNF^I97A^ and to understand its potential for clinical use.

## 4. Materials and Methods

### 4.1. Cell Lines

Chinese Hamster Ovary cells (CHO-S; Invitrogen, Carlsbad, CA, USA) were cultured in suspension in Power CHO-2CD medium (Lonza, Guangzhou, China), supplemented with Ultraglutamine-1 (4 mM; Lonza) and antibiotic-antimycotic (1%; AA; Gibco, New York, NY, USA). Human tumor cell lines A-375 (melanoma), A-431 (melanoma), HT-29 (adenocarcinoma), HT-1080 (fibrosarcoma), MDA-MB-231 (breast carcinoma), SKBR-3 (breast carcinoma), SKRC-52 (renal cell carcinoma), U-87 (glioma) and murine WEHI-164 (fibrosarcoma) were purchased from American Type Culture Collection (ATCC). Authentication of the cell line including check of post-freeze viability, growth properties and morphology, test for mycoplasma contamination, isoenzyme assay and sterility test were performed by the cell bank prior to shipment. Cells were cultured in the corresponding medium supplemented with Fetal Bovine Serum (10%; FBS; Invitrogen) and AA (1%) following the supplier’s protocol and kept in culture for no longer than 10 passages with a confluence lower than 90%.

### 4.2. Animals

Six to eight-week-old female BALB/c mice were obtained from Janvier. All the animal studies were conducted in accordance with Swiss animal welfare laws and regulations (license number ZH04/2018, granted by Veterinäramt des Kantons Zürich).

### 4.3. Structural Analysis of Human TNF and Design of Mutants

The structure of the wild type human TNF trimer was obtained from Protein Data Bank (PDB ID: 1TNF). The 3D structural model was used to identify residues at the monomer interface that are involved in trimerization by the formation of non-covalent intermolecular interactions. Residues involved in receptor binding interaction were discarded [37]. Selected residues were mutated in order to reduce or abrogate non-covalent interactions involved in the formation of trimeric TNF. The following mutants with a decreased potential in TNF trimerization were proposed: L19-TNF^Y59F^, L19-TNF^Q61S^, L19-TNF^Q61N^, L19-TNF^T72V^, L19-TNF^I97A^, L19-TNF^Q102N^, L19-TNF^Y119S^, L19-TNF^V123A^, L19-TNF^S147A^ and L19-TNF^Y151F^. Mutations were predicted and visualized using the mutagenesis wizard tool of the PyMOL molecular viewer (v1.8.2.2, Schrödinger LLC, New York, NY, USA). PyMOL was also used for the generation of figures and images.

### 4.4. Cloning, Expression and Biochemical Characterization of Proteins

Human TNF muteins were generated from the original construct L19-TNF^WT^, a fusion of the L19 antibody in scFv format to a TNF module at the C-terminal by the 17-amino-acid linker EF(SSSSG)_3_. HindIII, BamHI and NotI restriction sites were inserted for cloning purposes. The human TNF sequence was mutagenized using standard PCR with specific primers carrying each single point mutation (sequences described in the Supplementary Information Table S1) and cloned into the mammalian vector pcDNA3.1+ (Invitrogen) by BamHI/NotI restriction sites. All constructs were verified by DNA sequencing. The proteins were expressed by transient gene expression in CHO-S cells and purified from the cell culture supernatant to homogeneity by protein A (Sino Biological) chromatography, as described previously [47]. After dialyses into PBS pH 7.4, the quality of the proteins was assessed by SDS-PAGE, by size-exclusion chromatography on a Superdex 200 Increase 10/300 LGL column (ÄKTA FPLC, GE Healthcare, Arlington Heights, IL, USA) and LC-MS (Waters Xevo G2XS Q-TOF).

### 4.5. Immunofluorescence Experiments on Tumor Cell Lines

Different tumor cell lines were analyzed for EDB expression by immunofluorescence. Cells were grown to 80% confluence, detached with Trypsin-EDTA 0.05% (Thermo Fisher, Waltham, MA, USA) and resuspended to a final concentration of 4 × 10^5^ cells/mL in the corresponding medium. 24-well plates containing sterile 9 mm cover slips (Thermo Fisher, Waltham, MA, USA) were seeded with 500 μL of the cell resuspension. Once the full confluence was reached, the medium was removed and cells were fixed with an ice-cold fixation solution (Methanol/Acetone; 1:1) for 10 min and blocked in BSA (2%; Sigma Aldrich, Burlington, MA, USA) for 1 h. EDB was detected with the primary antibody L19 at 2 μg/mL in IgG format and with the secondary anti-human IgG conjugated with Alexa-488 (1:500; Invitrogen, Carlsbad, CA, USA). The KSF(IgG) antibody specific to the hen egg lysozyme, was used as the negative control [48]. Nuclei were counterstained with DAPI (1 μg/mL; Invitrogen) and cover slips were mounted with Dako fluorescent mounting medium (Agilent, Santa Clara, CA, USA). Staining was visualized using an Axioskop2 mot plus microscope (Zeiss, ETH, Switzerland) with a final magnification of 200X.

### 4.6. In Vitro Cytotoxicity Assays

The biocidal activity of the L19-TNF^WT^ and its mutants was determined in vitro on the human melanoma A-375 cell line. In order to distinguish variations in the direct killing of the proteins upon trimerization on demand, some wells were coated with 100 μL of sterile PBS containing 100 nM of EDB protein for 90 min at 37 °C. Wells were washed with PBS and 2 × 10^4^ cells/well were seeded in 96-well plates in DMEM medium (Gibco). After 24 h, new medium containing decreasing concentrations of the different proteins (1:10 dilution steps) was added to the cells in the presence of actinomycin D (Sigma; 2 μg/mL) to prevent the overgrowth of the culture. The cell viability was measured after 24 h by adding 20 μL of CellTiter 96^®^ AQueous One Solution Reagent (Promega, Madison, WI, USA) directly to the wells. The number of living cells was determined by measuring the absorbance after 4 h at 490 nm using a Spark multimode microplate reader (Tecan, Kawasaki City, Japan). Experiments were performed in triplicate and the results were expressed as the percentage of cell viability compared with controls (cells treated with actinomycin D only) and EC_50_ values with or without EDB coating were calculated for each mutant.

### 4.7. Affinity Measurements

Affinity measurements were performed by surface plasmon resonance using a BIAcore X100 (GE Healthcare) instrument using an EDB-coated CM5 chip at a density of 1500–2000 RU. L19-TNF^WT^, the non-depotentiated mutant L19-TNF^Q102N^ and the depotentiated version L19-TNF^I97A^ were diluted in a running buffer (PBS pH 7.4) before injection at a flow rate of 10 µL/min with a final increase of 340 RU. During the association phase, TNFR1 was injected for 30 s with a concentration range from 1 mM to 125 nM. Regeneration buffer HCl 10 mM was run over the chip for 5 min before each dilution. Independent chips were used for each protein and the data binding kinetics were calculated by global-fitting using the BIAcore Evaluation Software 3.2 RCI (GE Healthcare) applying a 1:1 Langmuir binding model.

### 4.8. Biodistribution Studies

The in vivo targeting of the mutant was assessed by quantitative biodistribution as previously described [49]. Radioiodinated L19-TNF^I97A^ (2 μg) was injected into the lateral tail vein of WEHI-164 tumor-bearing mice. Mice were killed 24 h after injection, and their organs were excised, weighed and radioactivity was measured using a Cobra γ counter (Packard, Meriden, CT, USA). The radioactivity of organs and tumors was expressed as the percentage of injected dose per gram of tissue (%ID/g ± standard error, *n* = 3) in a dot plot manner.

### 4.9. Immunofluorescence Studies on Tissue

Cryo-sections of 10 μm from non-treated mice bearing WEHI-164 tumor were fixed in ice-cold acetone for 10 min and blocked for 1 h in FBS/BSA (20%/2% solution in PBS). EDB expression was detected by using L19-TNF^WT^ or L19-TNF^I97A^ respectively (10 μg/mL in PBS/2% BSA). Alexa Fluor 488 protein-A conjugate (Thermo Fisher; 1:200) was used as a secondary detection antibody. Vessels were stained with a rat anti-mouse CD31 (BD Pharmigen; 1:200) and detected with a donkey anti-rat Alexa Fluor (Invitrogen; 1:500). Slides were mounted with Dako fluorescent mounting medium (Agilent) and sample representative pictures were taken with a 10x objective lens using an Axioskop2 mot plus microscope (Zeiss; ETH, Switzerland).

### 4.10. Therapy Experiments

WEHI-164 cells were grown to 80% confluence and detached with Trypsin-EDTA 0.05% (Life Technologies). Cells were washed, counted and re-suspended in HBSS to a final concentration of 1.7 × 10^7^ cells ml^−1^. Aliquots of 2.5 × 10^6^ cells (150 μL of a suspension) were injected s.c. in the right flank of the animal. Tumor volume was determined with the following formula: d^2^ × D × 0.5, where d and D are the short and long dimensions in millimeters, respectively, measured with a caliper. L19-TNF^WT^ and the mutant I97A were intravenously administered at the dose of 375 μg/Kg (schedule presented in Figure 5C, black arrows indicate injection days; *n* = 5). Toxicity induced by human TNF was monitored daily by checking the body weight and general appearance of the animals. A body weight loss higher than 10% was considered a severe consequence of the treatment. Animals were sacrificed when one or more termination criteria indicated by the experimental license were reached (e.g., body weight loss > 15%).

### 4.11. Statistics

Data was analyzed using Prism 7.0 (GraphPad Software, Inc., San Diego, CA, USA). A Student t test was used to assess the differences of cell viability between different experimental groups. A paired T-test was used to assess differences of the % ID/g between the tumor and healthy organs. Differences in tumor volume between therapeutic groups (until day 12, when n = 5) were evaluated with the two-way ANOVA followed by Bonferroni as post-test. *p* < 0.05 was considered statistically significant (* *p* < 0.05, ** *p* < 0.01, *** *p* < 0.001, **** *p* < 0.0001). Statistical analysis of the biodistribution and therapy experiments is documented in the Supplementary Information.

## Figures and Tables

**Table 1:** Calculated EC_50_ values for L19-TNF wild type and mutants.

	No EDB Coating (M)	100 nM EDB Coating (M)
L19-TNF^WT^	1.076 × 10^−10^	1.033 × 10^−10^
L19-TNF^Y59F^	4.172 × 10^−10^	9.501 × 10^−10^
L19-TNF^I97A^	2.273 × 10^−9^	2.254 × 10^−10^
L19-TNF^Q102N^	5.012 × 10^−11^	5.986 × 10^−11^
L19-TNF^Y119S^	3.245 × 10^−11^	6.417 × 10^−11^
L19-TNF^S147A^	1.261 × 10^−11^	1.58 × 10^−11^

Values were obtained by measuring viable cells after incubation with each molecule in the presence or absence of EDB coating (*n* = 3).

## Data Availability

The data presented in this study are available in this manuscript.

## Supplementary Materials

The following are available online at https://www.mdpi.com/article/10.3390/ijms221810020/s1.
