# Supplementary material for: An Attenuated Targeted-TNF Localizes to Tumors In Vivo and Regains Activity at the Site of Disease
Source: Int J Mol Sci. 2021 Sep 16;22(18):10020. doi: 10.3390/ijms221810020 (PMC8469155; doi:10.3390/ijms221810020)
Supplement: Supplementary file 1 [file ijms-22-10020-s001.zip › ijms-1366199-supplementary.pdf]

An attenuated targeted-TNF localizes to tumors *in vivo* and regains activity at the site of disease

Sheila Dakhel[1], Christian Lizak[1], Mattia Matasci[1], Jacqueline Mock[1], Alessandra Villa[1], Dario Neri[1,2], Samuele Cazzamalli[1]*

1) Philochem AG, Libernstrasse 3, CH-8112 Otelfingen (Switzerland)

2) Philogen SpA, Piazza La Lizza 7, 53100 Siena (Italy)

*) Corresponding author

phone: +41 43 544 88 19

fax: +41 43 544 88 09

samuele.cazzamalli@philogen.com

Table of Contents

Cloning of L19-TNF mutants **3**

Table S1. Nucleotide sequence of primers used for site-specific mutagenesis 3

Protein sequences of L19-TNF wild type and mutants **4**

Protein Characterization **8**

SDS-PAGE 8

Gel Filtration Analysis 9

Mass Spectrometry 9

Figure S1. Protein characterization of other L19-TNF mutants produced 10

Conditional in vitro cytotoxicity effect of L19-TNF mutants **11**

Table S4. IC50 values of *in vitro* killing assay for other L19-hTNF mutants 11

Fitting and calculated kinetics constants for L19-TNF^{WT} and its mutants **12**

Table S5. L19-TNF^{WT}-TNFR1 binding kinetics constants **12**

Table S6. L19-TNF^{I97A}-TNFR1 binding kinetics constants **12**

Biodistribution L19-TNF^{WT} **13**

Figure S2. *In vivo* biodistribution with radioiodinated L19-TNF^{WT} in BALB/c mice bearing WEHI-164 subcutaneous tumors. 13

In vivo MTD definition for L19-TNF products **14**

Figure S3. Systemic MTD for L19-TNF^{wt} and L19-TNF^{I97A} in mice 14

Statistical analysis of biodistribution experiments **15**

Table S7. Statistical analyses of %ID/g of L19-TNF^{I97A} (tumor vs. organs) 15

Statistical analysis of therapy experiments **16**

Table S8. Statistical analyses of therapy experiments 16

Body Weight Change (%) 16

Tumor Size (mg) 16

Cloning of L19-TNF mutants

Table S1. Nucleotide sequence of primers used for site-specific mutagenesis

	**Forward (5' → 3')**	**Reverse (5' → 3')**
**L19-TNF^{Y59F}**	CAGAGGGCCTGTACCTCATCTTCTCCCAGGTCCTCTTCAAGGG	CCCTTGAAGAGGACCTGGGAGAAGATGAGGTACAGGCCCTCTG
**L19-TNF^{I97A}**	AGGTCAACCTCCTCTCTGCCGCCAAGAGCCCCTGCCAGAGGGA	TCCCTCTGGCAGGGGCTCTTGGCGGCAGAGAGGAGGTTGACCT
**L19-TNF^{Q102N}**	CTGCCATCAAGAGCCCCTGCAATAGGGAGACCCCAGAGGGGGC	GCCCCCTCTGGGGTCTCCCTATTGCAGGGGCTCTTGATGGCAG
**L19-TNF^{Y119S}**	AGCCCTGGTATGAGCCCATCAGTCTGGGAGGGGTCTTCCAGCT	AGCTGGAAGACCCCTCCCAGACTGATGGGCTCATACCAGGGCT
**L19-TNF^{S147A}**	CGCGGATCCAGCTCTTCCGGCTCATCGTCCAGCGGCGTCAGATCATCTTCTCGAACC	TTTTCCTTTTGCGGCCGCTTATCACAGGGCAATGATCCCAAAGTAGACCTGCCCTGCCTC
**L19-TNF^{Q61S}**	GCCTGTACCTCATCTACTCCTCGGTCCTCTTCAAGGGCCAAGG	CCTTGGCCCTTGAAGAGGACCGAGGAGTAGATGAGGTACAGGC
**L19-TNF^{Q61N}**	GCCTGTACCTCATCTACTCCAACGTCCTCTTCAAGGGCCAAGG	CCTTGGCCCTTGAAGAGGACGTTGGAGTAGATGAGGTACAGGC
**L19-TNF^{T72V}**	AGGGCCAAGGCTGCCCCTCCGTCCATGTGCTCCTCACCCACAC	GTGTGGGTGAGGAGCACATGGACGGAGGGGCAGCCTTGGCCCT
**L19-TNF^{V123A}**	AGCCCATCTATCTGGGAGGGGCCTTCCAGCTGGAGAAGGGTGA	TCACCCTTCTCCAGCTGGAAGGCCCCTCCCAGATAGATGGGCT
**L19-TNF^{Y151F}**	CGCGGATCCAGCTCTTCCGGCTCATCGTCCAGCGGCGTCAGATCATCTTCTCGAACC	TTTTCCTTTTGCGGCCGCTTATCACAGGGCAATGATCCCAAAGAAGACCTGCCCAGA

Protein sequences of L19-TNF wild type and mutants

L19-hTNF^{WT}

EVQLLESGGGLVQPGGSLRLSCAASGFTFSSFSMSWVRQAPGKGLEWVSSISGSSGTTYY

ADSVKGRFTISRDNSKNTLYLQMNSLRAEDTAVYYCAKPFPYFDYWGQGTLVTVSSGDGS

SGGSGGASEIVLTQSPGTLSLSPGERATLSCRASQSVSSSFLAWYQQKPGQAPRLLIYYAS

SRATGIPDRFSGSGSGTDFTLTISRLEPEDFAVYYCQQTGRIPPTFGQGTKVEIKEFSSSSG

SSSSGSSSSGVRSSSRTPSDKPVAHVVANPQAEGQLQWLNRRANALLANGVELRDNQLV

VPSEGLYLIYSQVLFKGQGCPSTHVLLTHTISRIAVSYQTKVNLLSAIKSPCQRETPEGAEAK

PWYEPIYLGGVFQLEKGDRLSAEINRPDYLDFAESGQVYFGIIAL

L19-hTNF^{Y59F}

EVQLLESGGGLVQPGGSLRLSCAASGFTFSSFSMSWVRQAPGKGLEWVSSISGSSGTTYY

ADSVKGRFTISRDNSKNTLYLQMNSLRAEDTAVYYCAKPFPYFDYWGQGTLVTVSSGDGS

SGGSGGASEIVLTQSPGTLSLSPGERATLSCRASQSVSSSFLAWYQQKPGQAPRLLIYYAS

SRATGIPDRFSGSGSGTDFTLTISRLEPEDFAVYYCQQTGRIPPTFGQGTKVEIKEFSSSSG

SSSSGSSSSGVRSSSRTPSDKPVAHVVANPQAEGQLQWLNRRANALLANGVELRDNQLV

VPSEGLYLIFSQVLFKGQGCPSTHVLLTHTISRIAVSYQTKVNLLSAIKSPCQRETPEGAEAK

PWYEPIYLGGVFQLEKGDRLSAEINRPDYLDFAESGQVYFGIIAL

L19-hTNF^{Q61S}

EVQLLESGGGLVQPGGSLRLSCAASGFTFSSFSMSWVRQAPGKGLEWVSSISGSSGTTYY

ADSVKGRFTISRDNSKNTLYLQMNSLRAEDTAVYYCAKPFPYFDYWGQGTLVTVSSGDGS

SGGSGGASEIVLTQSPGTLSLSPGERATLSCRASQSVSSSFLAWYQQKPGQAPRLLIYYAS

SRATGIPDRFSGSGSGTDFTLTISRLEPEDFAVYYCQQTGRIPPTFGQGTKVEIKEFSSSSG

SSSSGSSSSGVRSSSRTPSDKPVAHVVANPQAEGQLQWLNRRANALLANGVELRDNQLV

VPSEGLYLIYSSVLFKGQGCPSTHVLLTHTISRIAVSYQTKVNLLSAIKSPCQRETPEGAEAK

PWYEPIYLGGVFQLEKGDRLSAEINRPDYLDFAESGQVYFGIIAL

L19-hTNF^{Q61N}

EVQLLESGGGLVQPGGSLRLSCAASGFTFSSFSMSWVRQAPGKGLEWVSSISGSSGTTYY

ADSVKGRFTISRDNSKNTLYLQMNSLRAEDTAVYYCAKPFPYFDYWGQGTLVTVSSGDGS

SGGSGGASEIVLTQSPGTLSLSPGERATLSCRASQSVSSSFLAWYQQKPGQAPRLLIYYAS

SRATGIPDRFSGSGSGTDFTLTISRLEPEDFAVYYCQQTGRIPPTFGQGTKVEIKEFSSSSG

SSSSGSSSSGVRSSSRTPSDKPVAHVVANPQAEGQLQWLNRRANALLANGVELRDNQLV

VPSEGLYLIYSNVLFKGQGCPSTHVLLTHTISRIAVSYQTKVNLLSAIKSPCQRETPEGAEAK

PWYEPIYLGGVFQLEKGDRLSAEINRPDYLDFAESGQVYFGIIAL

L19-hTNF^{T72V}

EVQLLESGGGLVQPGGSLRLSCAASGFTFSSFSMSWVRQAPGKGLEWVSSISGSSGTTYY

ADSVKGRFTISRDNSKNTLYLQMNSLRAEDTAVYYCAKPFPYFDYWGQGTLVTVSSGDGS

SGGSGGASEIVLTQSPGTLSLSPGERATLSCRASQSVSSSFLAWYQQKPGQAPRLLIYYAS

SRATGIPDRFSGSGSGTDFTLTISRLEPEDFAVYYCQQTGRIPPTFGQGTKVEIKEFSSSSG

SSSSGSSSSGVRSSSRTPSDKPVAHVVANPQAEGQLQWLNRRANALLANGVELRDNQLV

VPSEGLYLIYSQVLFKGQGCPSVHVLLTHTISRIAVSYQTKVNLLSAIKSPCQRETPEGAEAK

PWYEPIYLGGVFQLEKGDRLSAEINRPDYLDFAESGQVYFGIIAL

L19-hTNF^{I97A}

EVQLLESGGGLVQPGGSLRLSCAASGFTFSSFSMSWVRQAPGKGLEWVSSISGSSGTTYY

ADSVKGRFTISRDNSKNTLYLQMNSLRAEDTAVYYCAKPFPYFDYWGQGTLVTVSSGDGS

SGGSGGASEIVLTQSPGTLSLSPGERATLSCRASQSVSSSFLAWYQQKPGQAPRLLIYYAS

SRATGIPDRFSGSGSGTDFTLTISRLEPEDFAVYYCQQTGRIPPTFGQGTKVEIKEFSSSSG

SSSSGSSSSGVRSSSRTPSDKPVAHVVANPQAEGQLQWLNRRANALLANGVELRDNQLV

VPSEGLYLIYSQVLFKGQGCPSTHVLLTHTISRIAVSYQTKVNLLSAAKSPCQRETPEGAEA

KPWYEPIYLGGVFQLEKGDRLSAEINRPDYLDFAESGQVYFGIIAL

L19-hTNF^{Q102N}

EVQLLESGGGLVQPGGSLRLSCAASGFTFSSFSMSWVRQAPGKGLEWVSSISGSSGTTYY

ADSVKGRFTISRDNSKNTLYLQMNSLRAEDTAVYYCAKPFPYFDYWGQGTLVTVSSGDGS

SGGSGGASEIVLTQSPGTLSLSPGERATLSCRASQSVSSSFLAWYQQKPGQAPRLLIYYAS

SRATGIPDRFSGSGSGTDFTLTISRLEPEDFAVYYCQQTGRIPPTFGQGTKVEIKEFSSSSG

SSSSGSSSSGVRSSSRTPSDKPVAHVVANPQAEGQLQWLNRRANALLANGVELRDNQLV

VPSEGLYLIYSQVLFKGQGCPSTHVLLTHTISRIAVSYQTKVNLLSAIKSPCNRETPEGAEAK

PWYEPIYLGGVFQLEKGDRLSAEINRPDYLDFAESGQVYFGIIAL

L19-hTNF^{Y119S}

EVQLLESGGGLVQPGGSLRLSCAASGFTFSSFSMSWVRQAPGKGLEWVSSISGSSGTTYY

ADSVKGRFTISRDNSKNTLYLQMNSLRAEDTAVYYCAKPFPYFDYWGQGTLVTVSSGDGS

SGGSGGASEIVLTQSPGTLSLSPGERATLSCRASQSVSSSFLAWYQQKPGQAPRLLIYYAS

SRATGIPDRFSGSGSGTDFTLTISRLEPEDFAVYYCQQTGRIPPTFGQGTKVEIKEFSSSSG

SSSSGSSSSGVRSSSRTPSDKPVAHVVANPQAEGQLQWLNRRANALLANGVELRDNQLV

VPSEGLYLIYSQVLFKGQGCPSTHVLLTHTISRIAVSYQTKVNLLSAIKSPCQRETPEGAEAK

PWYEPISLGGVFQLEKGDRLSAEINRPDYLDFAESGQVYFGIIAL

L19-hTNF^{V123A}

EVQLLESGGGLVQPGGSLRLSCAASGFTFSSFSMSWVRQAPGKGLEWVSSISGSSGTTYY

ADSVKGRFTISRDNSKNTLYLQMNSLRAEDTAVYYCAKPFPYFDYWGQGTLVTVSSGDGS

SGGSGGASEIVLTQSPGTLSLSPGERATLSCRASQSVSSSFLAWYQQKPGQAPRLLIYYAS

SRATGIPDRFSGSGSGTDFTLTISRLEPEDFAVYYCQQTGRIPPTFGQGTKVEIKEFSSSSG

SSSSGSSSSGVRSSSRTPSDKPVAHVVANPQAEGQLQWLNRRANALLANGVELRDNQLV

VPSEGLYLIYSQVLFKGQGCPSTHVLLTHTISRIAVSYQTKVNLLSAIKSPCQRETPEGAEAK

PWYEPIYLGGAFQLEKGDRLSAEINRPDYLDFAESGQVYFGIIAL

L19-hTNF^{S147A}

EVQLLESGGGLVQPGGSLRLSCAASGFTFSSFSMSWVRQAPGKGLEWVSSISGSSGTTYY
ADSVKGRFTISRDNSKNTLYLQMNSLRAEDTAVYYCAKPFPYFDYWGQGTLVTVSSGDGS
SGGSGGASEIVLTQSPGTLSLSPGERATLSCRASQSVSSSFLAWYQQKPGQAPRLLIYYAS
SRATGIPDRFSGSGSGTDFTLTISRLEPEDFAVYYCQQTGRIPPTFGQGTKVEIKEFSSSSG
SSSSGSSSSGVRSSSRTPSDKPVAHVVANPQAEGQLQWLNRRANALLANGVELRDNQLV
VPSEGLYLIYSQVLFKGQGCPSTHVLLTHTISRIAVSYQTKVNLLSAIKSPCQRETPEGAEAK
PWYEPIYLGGVFQLEKGDRLSAEINRPDYLDFAEAGQVYFGIIAL

L19-hTNF^{Y151F}

EVQLLESGGGLVQPGGSLRLSCAASGFTFSSFSMSWVRQAPGKGLEWVSSISGSSGTTYY
ADSVKGRFTISRDNSKNTLYLQMNSLRAEDTAVYYCAKPFPYFDYWGQGTLVTVSSGDGS
SGGSGGASEIVLTQSPGTLSLSPGERATLSCRASQSVSSSFLAWYQQKPGQAPRLLIYYAS
SRATGIPDRFSGSGSGTDFTLTISRLEPEDFAVYYCQQTGRIPPTFGQGTKVEIKEFSSSSG
SSSSGSSSSGVRSSSRTPSDKPVAHVVANPQAEGQLQWLNRRANALLANGVELRDNQLV
VPSEGLYLIYSQVLFKGQGCPSTHVLLTHTISRIAVSYQTKVNLLSAIKSPCQRETPEGAEAK
PWYEPIYLGGVFQLEKGDRLSAEINRPDYLDFAESGQVFFGIIAL

Protein Characterization

SDS-PAGE

Protein samples were diluted to 0.2-0.3 mg/mL in PBS and mixed with either reducing or non-reducing 5x Loading buffer. Samples were denatured 5’ at 95 °C and loaded on NuPAGE 4-12% Bis-Tris Gel (Novex™ by Life Technologies). 1x MES NuPAGE (Novex™ by Life Technologies) was used as running buffer and electrophoresis was performed at 180 V, 110 mA for 1h. Gel was rinsed with deionized water and stained in Coomassie blue for 15-20’ on an orbital shaker. Staining solution was discarded and the gel was rinsed 3 times with deionized water and immerged in destaining solution (10% acetic acid/30% methanol/mQ water) for 3-12h on an orbital shaker. Destaining solution was discarded and recycled, gel was rinsed with deionized water and a picture of the gel was taken. Recipes for the 5X Loading buffer and Coomassie blue stain are as described in Table S2 and S3:

**100mL, 5X non-red Loading Buffer**	
Tris-HCl (250mM, pH 6.8)	20.8 mL
Glycerol	33.3 mL
SDS	6.6 g
Bromophenol blue	66 mg
mQ water	up to 100 mL

Table S2**.** 5X Loading buffer recipe

**1L Coomassie blue**	
PlusOne Coomassie PhastGel Blue R-350	2 tablets
Methanol	400 mL
Acetic Acid	100 mL
mQ water	500 mL

Table S3**.** Coomassie blue recipe

Gel Filtration Analysis

100 µL of diluted sample (final concentration 0.1-0.5 mg/mL) were loaded on FPLC (Äkta, GE Healthcare) and protein were separated by a Superdex200 Increase 10/300 GL column (GE Healthcare) previously equilibrated with 1 CV PBS, using PBS as mobile phase at a flow rate of 0.6 mL/min (column pressure limit set at 5 MPa). Proteins were detected by an UV-detector at a wavelength of 280 nm.

Mass Spectrometry

Samples were diluted to about 0.1 mg/mL and LC-MS was performed on a Waters Xevo G2XS Qtof instrument (ESI-ToF-MS) coupled to a Waters Acquity UPLC H-Class System using a 2.1 × 50 mm Acquity BEH300 C4 1.7 µm column (Waters). 0.1% FA in water (solvent A) and 0.1% FA in MeCN (solvent B) were used as mobile phase at a flow rate of 0.4 mL/min. Gradient was programmed as follows: after 1.5 min isocratic with 95% solvent A, stepwise change from 95% solvent A to 95% solvent B in 4.5 min (10% increase every 0.5 min), back to 95% solvent A in 0.5 min, linearly to 95% solvent B and back to 95% solvent A in 2.25 min (last step repeated twice).

Figure S1. Protein characterization of other L19-TNF mutants produced

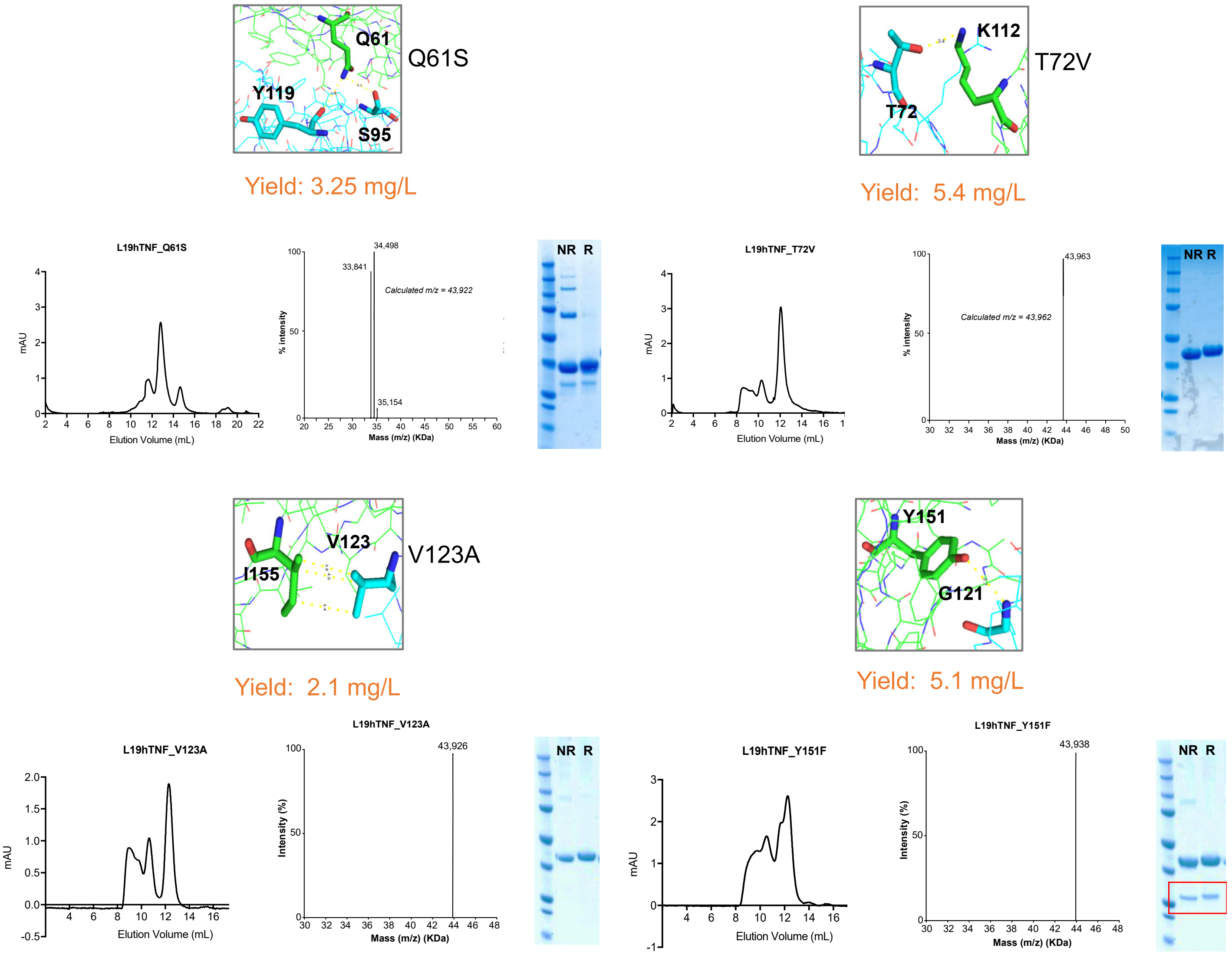

<u>Conditional *in vitro* cytotoxicity effect of L19-TNF mutants</u>

Table S4. IC50 values of *in vitro* killing assay for other L19-hTNF mutants

	**No EDB coating (M)**	**100nM EDB coating (M)**
**L19-TNF^{wt}**	6.219e-011	1.408e-011
**L19-TNF^{T72V}**	7.768e-011	4.772e-011
**L19-TNF^{V123A}**	1.801e-011	3.298e-011
**L19-TNF^{Y151F}**	7.127e-012	2.229e-011

Fitting and calculated kinetics constants for L19-TNF^{WT} and its mutants

The data binding kinetics were calculated by global-fitting using the BIAcore Evaluation Software 3.2 RCI (GE Healthcare) applying a 1:1 Langmuir binding model.

Table S5. L19-TNF^{WT}-TNFR1 binding kinetics constants

	ka (1/Ms)	kd (1/s)	Rmax (RU)	RI (RU)	Conc of analyte	KA (1/M)	KD (M)	Req (RU)	kobs (1/s)	Chi2
										0.378
Subtracted Fc=2-1	9.09e4	1.07e-3	74.2	0.97	250n	8.5e7	1.18e-8	70.9	0.0238	
Subtracted Fc=2-1	1.08e5	1.11e-3	88.7	-0.913	500n	9.72e7	1.03e-8	86.9	0.0551	
Subtracted Fc=2-1	1.1e5	1.58e-3	101	-3.48	1000n	6.98e7	1.43e-8	99.9	0.112	

Table S6. L19-TNF^{I97A}-TNFR1 binding kinetics constants

	ka (1/Ms)	kd (1/s)	Rmax (RU)	RI (RU)	Conc of analyte	KA (1/M)	KD (M)	Req (RU)	kobs (1/s)	Chi2
										0.154
Subtracted Fc=2-1	1.18e5	0.0606	73.1	-5.68	250n	1.95e6	5.13e-7	24	0.0902	
Subtracted Fc=2-1	1.4e5	0.0519	65.6	-2.07	500n	2.71e6	3.69e-7	37.7	0.122	
Subtracted Fc=2-1	1.49e5	0.0529	71.1	-2.77	1000n	2.83e6	3.54e-7	52.5	0.202	

Biodistribution L19-TNF^{WT}

Radioiodinated L19-TNF^{WT} (2 µg) was injected into the lateral tail vein of a single mice bearing WEHI-164 tumor. The animal was killed 24 h after injection, and organs were excised, weighed and radioactivity was measured using a Cobra γ counter (Packard, Meriden, CT, USA). The radioactivity of the organ and the tumor was expressed as the percentage of injected dose per gram of tissue (%ID/g ± standard error, n = 1).

Figure S2. *In vivo* biodistribution with radioiodinated L19-TNF^{WT} in BALB/c mice bearing WEHI-164 subcutaneous tumors.

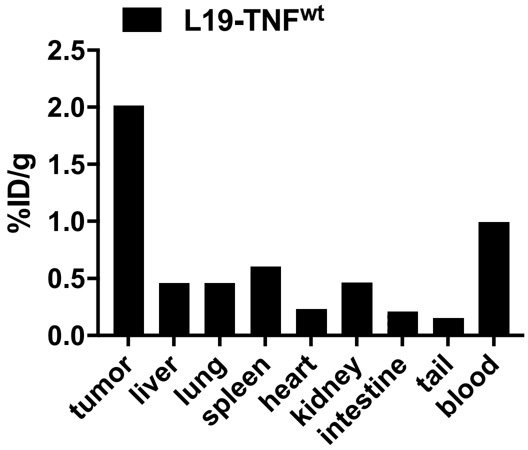

In vivo MTD definition for L19-TNF products

A dose escalation study with L19-TNF wild type and the mutant I97A was performed to define the corresponding maximum tolerated dose (MTD) of each molecule. Mice were subcutaneously-grafted with WEHI-164 tumor (2.5 × 10^6 cells/animal). Tumors were grown up to 100 mm^3 before the starting the injections. Three different injections were given to the mouse receiving 7.5 μg while a single intravenous injection for the higher doses could be administered due to very severe toxicity. Body weight loss (BWL) of the animals was measured daily (n = 1/dose/molecule) in order to determine toxicity induced by the molecules (represented in the graph by + = <15% BWL or ++ = > 15% BWL). More than 15% of BWL was a termination criteria of the study.

Figure S3. Systemic MTD for L19-TNF^{wt} and L19-TNF^{I97A} in mice

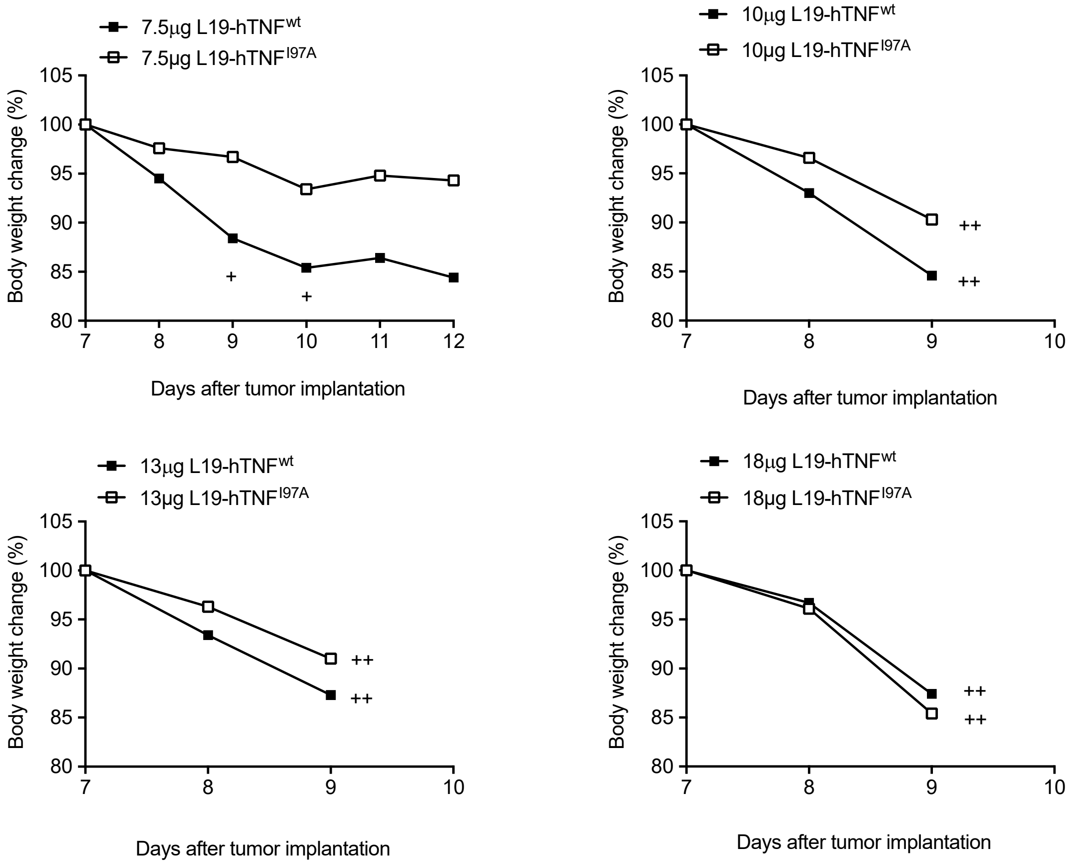

Statistical analysis of biodistribution experiments

Differences in %ID/g of tumor and normal organs were compared using the t-test analysis with one-tailed p-value of Graphpad Prism 7 (La Jolla, CA, USA).

Table S7. Statistical analyses of %ID/g of L19-TNF^{I97A} (tumor vs. organs)

**%ID/g L19-TNF^{I97A}**	
Tumor-Liver	$p < 0.05$
Tumor-Lung	$p < 0.05$
Tumor-Spleen	$p < 0.05$
Tumor-Heart	$p < 0.05$
Tumor-Kidney	$p < 0.05$
Tumor-Intestine	$p < 0.01$
Tumor-Tail	$p < 0.01$
Tumor-Blood	non-significant differences

Statistical analysis of therapy experiments

Differences in tumor volume and body weight between therapeutic groups were compared using the two-way ANOVA analysis with Bonferroni post-test of Graphpad Prism 7 (La Jolla, CA, USA). Days are counted after tumor implantation (*$p < 0.05$, **$p < 0.01$, ***$p < 0.001$, ****$p < 0.0001$).

Table S8. Statistical analyses of therapy experiments.

Body Weight Change (%)

L19-TNF^{wt} (375 µg/Kg) vs L19-TNF^{I97A} (375 µg/Kg).

day 5	non-significant differences
day 6	non-significant differences
day 7	$p < 0.001$
day 8	$p < 0.0001$
day 9	$p < 0.0001$
day 10	$p < 0.01$
day 11	$p < 0.01$
day 12	$p < 0.05$

Tumor Size (mg)

L19-TNF^{wt} (375 µg/Kg) vs L19-TNF^{I97A} (375 µg/Kg).

From day 5 until day 12	non-significant differences
